# Vismodegib for treatment of periocular basal cell carcinoma – 6-year experience from a tertiary cancer center^☆^^☆☆^

**DOI:** 10.1016/j.abd.2021.04.012

**Published:** 2021-09-11

**Authors:** Catarina Xavier, Edgar Lopes, Catarina Bexiga, Cecília Moura, Emanuel Gouveia, Ana Filipa Duarte

**Affiliations:** a Department of Ophthalmology, Centro Hospitalar Universitário de Lisboa Central, Lisboa, Portugal; b Department of Oncology, Instituto Português de Oncologia Francisco Gentil, Lisboa, Portugal; c Department of Dermatology, Instituto Português de Oncologia Francisco Gentil, Lisboa, Portugal; d Department of Ophthalmology, Instituto Português de Oncologia Francisco Gentil, Lisboa, Portugal

**Keywords:** Hedgehog proteins, Skin neoplasms, Carcinoma, basal cell

## Abstract

**Background:**

The treatment of advanced periocular basal cell carcinomas becomes a challenge as surgery may involve highly mutilating procedures. Vismodegib is the first selective hedgehog inhibitor approved for the treatment of locally advanced tumors or metastatic disease.

**Objective:**

Analyze the results of treatment with vismodegib for advanced periocular basal cell carcinomas in a real-life setting of a reference center between 2014 and 2020.

**Methods:**

Retrospective longitudinal study. The patient's demographic profile, comorbidities, tumor characteristics, and treatment outcomes were analyzed.

**Results:**

A total of 13 patients were included. Median follow-up and treatment duration were 15.9 and 10.5 months, respectively. Objective clinical response rate was 76.9%: 30.8% had a complete response and 46.2% a partial response. The median duration of response was 13 months. Progressive disease was observed in 38.5% of cases, with a median of 19 months after the beginning of treatment. Eighty-four percent of the patients had at least one adverse event, and 61.54% needed to interrupt treatment temporarily or permanently to increase tolerability.

**Study limitations:**

Being a retrospective study in a real-life setting, the evaluation of objective clinical response was subjective to physician appreciation.

**Conclusion:**

Vismodegib is a safe and effective treatment for locally advanced basal cell carcinoma. To prevent recurrences, the drug should be used continually when tolerated. The role of neoadjuvant vismodegib before surgery is being investigated and might add an important step in searching for a definitive treatment for these cases.

## Introduction

Basal Cell Carcinoma (BCC) is the most common type of cancer in the periocular area. It is a slowly growing tumor but can cause extensive local destruction of skin, muscle, or even bone.^1^ These Locally Advanced Basal Cell Carcinomas (laBCC) arise either from early lesions that have not been treated or from a recurrence of aggressive subtypes of BCC. When localized to the periocular area they can cause substantial morbidity. Metastatic Basal Cell Carcinomas (mBCC) are extremely rare.^2^, ^3^

In most cases, BCC can be treated with surgery, cryotherapy, or laser ablation. Other non-surgical therapies such as radiotherapy, photodynamic therapy, and topical treatment with imiquimod or 5-fluorouracil may be used in locally circumscribed BCC.^2^ The treatment of periocular laBCC becomes a challenge since extensive surgery and radiotherapy may comprise considerable morbidity.

The majority of sporadic BCC show mutations that initiate the sonic hedgehog pathway and cause uncontrolled cell proliferation.^4^ Being the main driver in BCC pathogenesis and progression, the Hedgehog pathway represents a critical therapeutic target.^4^ Vismodegib is the first selective Hedgehog inhibitor approved for the treatment of laBCC or mBCC in adults who cannot be treated with surgery or radiotherapy.^5^

This work aims to describe the experience from a tertiary cancer center using vismodegib for advanced periocular BCCs.

## Methods

We conducted a retrospective longitudinal consecutive study of the patients who started vismodegib (150 mg/day) for periocular BCC in a tertiary cancer center (Instituto Português de Oncologia Francisco Gentil, Lisbon) between July 2014 and January 2020. The patient's demographic profile, treatment outcomes, and adverse events were analyzed.

The primary outcome was defined as an Objective clinical Response Rate (ORR), which was the sum of the complete and partial response rates. We considered Complete Response (CR) the absence of clinical tumor and Partial Response (PR) the decrease in the tumor's size without its disappearance. Stable disease (SD) was considered when vismodegib could cease the tumor's progression, but there was no decrease in its size. When the tumor showed characteristics of regrowth, it was considered Progressive Disease (PD).

Other variables evaluated were the duration of treatment, the Duration of Response (DOR) – the time from the beginning of the therapy to PD, death, or the last control date when still responding. Time and cause of death were also registered.

Lastly, the authors also studied the drug's tolerability by analyzing each patient's adverse events and if they led to treatment interruption or discontinuation.

## Results

Of a total of 26 patients who received vismodegib treatment, 13 had periocular laBCC and were included in the present study: 6 were males and 7 females, with a mean age of 73 (54–96) years old at the beginning of the treatment (Table 1).

**Table 1:** Demographic and treatment-related data of the patients included in this case series.

Demographic and treatment-related data										
n	Age	Gender	Duration of treatment (months)	Objective response	DOR (months)	PD	Currently under	Adverse events	Last follow-up (months)	Timing and cause of death
1	83	F	30.8	SD	30.8	Yes	No	Fatigue	32.7	54.30 unknown
2	62	M	2.7	SD	2.6	No	No	–	2.7	2.70 Septic shock
3	78	F	24.1	CR	24	No	Yes	Muscle spasms, alopecia, dysgeusia, weight loss, fatigue, nausea, anorexia, arthralgias	24.1	–
4	67	M	6	CR	19	Yes	Yes	Muscle spasms, dysgeusia, weight loss, fatigue	15.9	–
5	61	M	10.5	CR	13	Yes	Yes	Muscle spasms, alopecia, dysgeusia, fatigue, nausea, diarrhea	21.9	–
6	82	F	6.5	PR	5.5	Yes	No	Fatigue, anorexia	6.5	7.6 Progressive disease
7	70	M	3.8	CR	11.8	No	No	Muscle spasms, alopecia, dysgeusia, weight loss, fatigue	11.8	11.8 unknown
8	76	F	41.2	PR	41.2	No	Yes	Muscle spasms, alopecia, fatigue, nausea, anorexia	41.2	–
9	54	F	32.8	SD	32.5	Yes	No	Muscle spasms, alopecia, dysgeusia, fatigue, nausea, anorexia	48.8	–
10	65	F	12.9	PR	52.5	No	No	Muscle spasms, alopecia, dysgeusia, weight loss	53.5	–
11	96	F	5.1	PR	5.1	No	Yes	–	5.1	–
12	71	M	9.3	PR	9.2	No	Yes	Muscle spasms	9.3	–
13	84	M	10.5	PR	10.5	No	Yes	Muscle spasms	10.5	–

M, Male; F, Female; SD, stable disease; CR, Complete Response; PR, Partial Response; PD, progressive disease; DOR, duration of response.

The treatment was administered during a median of 10.5 (2.7–41.2) months. ORR was 76.9%: 4 (30.8%) patients with CR and 6 (46.2%) with PR. SD was observed in 3 cases (23.1%). The median DOR was 13 (2.6–52.5) months.

With a median follow-up time of 15.9 (2.7–53.5) months, the total PD rate was 38.5% (5 patients) in a median time of 19 (5.5–32.5) months after starting vismodegib. Two of these cases were patients with SD, with a mean DOR of 32 months; one of them was under intermittent treatment due to poor tolerability and died of an unknown cause 2 years after PD. Another case of PD was a PR who had a DOR of 5.5 months. This patient died due to complications of the BCC 2 months after PD appearance. The other 2 cases with PD had an initial clinical CR, with a mean DOR of 16 months; PD occurred after discontinuation of the treatment due to toxicity – vismodegib was later reintroduced with PR. One of these patients underwent a biopsy while on CR that didn't show neoplastic cells.

Concerning the 4 cases with clinical CR, 2 had PD, as described above, one is still under treatment (after 24 months) without PD, and the remaining one stopped the drug after undergoing surgery that didn't show neoplastic cells on the histology. The latter died of other medical comorbidities 10 months after stopping vismodegib, without signs of PD.

Considering the safety profile and treatment tolerance, most patients (84.6%) had at least one adverse event, the most common being muscle spasms (69%), fatigue (61.5%), alopecia (46.2%), and dysgeusia (46.2%). Despite being mostly mild toxicity, 1 patient (7,68%) discontinued the treatment permanently, and 7 patients (53.85%) needed to interrupt vismodegib to increase tolerability. No patients had signs of rhabdomyolysis nor developed squamous cell carcinoma. During follow-up, four patients died, only 1 of them related to PD.

## Discussion

The treatment of advanced forms of BCC is a challenge, and until recent years limited to surgery and/or radiation therapy. Vismodegib has unquestionably shifted the approach to these cases. Two international, multicenter clinical trials showed ORRs of 68.5% and 60.3% for laBCC 4, 6 with the use of this drug. These results are inferior to the ORR of 76.9% reported in this study, which is probably overestimated due to less strict and more subjective clinical criteria used in real-life practice.

The most common adverse events described in the literature are muscle spasm, dysgeusia, and alopecia,^4^ which is in accordance with the present study’s results. These were generally mild, however very frequent, and in a not negligible number of cases lead to the discontinuation of treatment.

Temporary interruption of vismodegib (on and off regimen) is a commonly used strategy that allows patients to remain on treatment without reducing efficacy.^4^ The primary analysis of the MIKIE study,^7^ which compared two intermittent regimens with 8 weeks pauses, showed that the interruption of treatment did not compromise the activity of vismodegib. Both regimens controlled the disease during the entire treatment period in most patients.^7^ An exploratory analysis of the STEVIE study^4^ also demonstrated that an increased treatment interruption was associated with increased median treatment duration and overall response rate.^4^ Therefore, the interruption of vismodegib has been included in a consensus recommendation of treatment strategies in patients with advanced BCC.^7^

The authors noticed that 60% of the PD cases in the present study occurred during periods of treatment interruption superior to 8 weeks. The authors agree that as long as the adverse events are tolerated, vismodegib can be taken on a continuous regimen and that intermittent regimens should limit the intervals without treatment to a maximum of 2 months, if possible. Importantly, in poor tolerability cases, an intermittent regimen should always be discussed before deciding to stop the treatment. The authors believe that the study’s outcomes using vismodegib could be improved with a stricter protocol for cases that require an intermittent regimen.

A histologic cure is not guaranteed with the Hedgehog inhibitor therapy alone, even when complete clinical remission is obtained. Some authors suggest that the discontinuation of the drug without additional surgery may leave the residual or noncontiguous tumor, potentially resistant to treatment.^1^ The risk of subsequent tumor regrowth, which may be more aggressive or difficult to treat, supports the use of these drugs in association with other modalities of treatment.^1^, ^8^ MS Ally et al.^8^ showed that the use of vismodegib as neoadjuvant therapy for at least 3 months before surgery presented an overall benefit in reducing the tumor area and surgical defect size. However, if skip areas of apparent tumor-free tissue alternating with clinical BCC develop, this modality could potentially lead to delayed tumor recurrence in the supposedly treated areas.^1^, ^8^ This could explain the disease's recurrence in the study’s patient with CR whose biopsy didn't show malignant cells. Such tumors would require wide-margin surgery to optimize local control and minimize the recurrence risk.^1^, ^8^

## Conclusion

The present study’s results support the evidence that vismodegib is a safe and effective treatment for laBCC; nevertheless, further studies are needed to establish the efficacy of vismodegib, and most importantly, its place in the neoadjuvant setting. Its role in unresectable laBCC as a bridge to surgery seems to be an important step toward the definitive treatment of these challenging cases.

## Financial support

None declared.

## Authors’ contributions

Catarina Xavier: Approval of the final version of the manuscript; critical literature review; data collection, analysis, and interpretation; effective participation in research orientation; preparation and writing of the manuscript; statistical analysis; study conception and planning.

Edgar Lopes: Approval of the final version of the manuscript; critical literature review; data collection, analysis, and interpretation; effective participation in research orientation; preparation and writing of the manuscript.

Catarina Bexiga: Approval of the final version of the manuscript; critical literature review; data collection, analysis, and interpretation; effective participation in research orientation; intellectual participation in propaedeutic and/or therapeutic management of studied cases; study conception and planning.

Cecília Moura: Approval of the final version of the manuscript; intellectual participation in propaedeutic and/or therapeutic management of studied cases; manuscript critical review; study conception and planning.

Cecília Moura: Approval of the final version of the manuscript; intellectual participation in propaedeutic and/or therapeutic management of studied cases; manuscript critical review; study conception and planning.

Ana Filipa Duarte: Approval of the final version of the manuscript; intellectual participation in propaedeutic and/or therapeutic management of studied cases; manuscript critical review; study conception and planning.

## Conflicts of interest

None declared.
